# Identification of lysine methylation in the core GTPase domain by GoMADScan

**DOI:** 10.1371/journal.pone.0219436

**Published:** 2019-08-07

**Authors:** Hirofumi Yoshino, Guowei Yin, Risa Kawaguchi, Konstantin I. Popov, Brenda Temple, Mika Sasaki, Satoshi Kofuji, Kara Wolfe, Kaori Kofuji, Koichi Okumura, Jaskirat Randhawa, Akshiv Malhotra, Nazanin Majd, Yoshiki Ikeda, Hiroko Shimada, Emily Rose Kahoud, Sasson Haviv, Shigeki Iwase, John M. Asara, Sharon L. Campbell, Atsuo T. Sasaki

**Affiliations:** 1 Division of Hematology and Oncology, Department of Internal Medicine, University of Cincinnati College of Medicine, Cincinnati, Ohio, United States of America; 2 Department of Biochemistry and Biophysics and Lineberger Comprehensive Cancer Center, University of North Carolina School of Medicine, Chapel Hill, North Carolina, United States of America; 3 Department of Computational Biology and Medical Sciences, Graduate School of Frontier Sciences, University of Tokyo, Kashiwa, Chiba, Japan; 4 University of North Carolina, R. L. Juliano Structural Bioinformatics Core Facility, Chapel Hill, North Carolina, United States of America; 5 Department of Neurology, University of Cincinnati College of Medicine, Cincinnati, Ohio, United States of America; 6 Harvard Medical School, Department of Medicine and Beth Israel Deaconess Medical Center, Division of Signal Transduction, Boston, Massachusetts, United States of America; 7 Department of Human Genetics, University of Michigan, 5815 Medical Science II, Ann Arbor, Michigan, United States of America; 8 Department of Cancer Biology, University of Cincinnati College of Medicine, Ohio, United States of America; 9 Department of Neurosurgery, Brain Tumor Center at UC Gardner Neuroscience Institute, Cincinnati, Ohio, United States of America; 10 Institute for Advanced Biosciences, Keio University, Tsuruoka, Yamagata, Japan; Universität Stuttgart, GERMANY

## Abstract

RAS is the founding member of a superfamily of GTPases and regulates signaling pathways involved in cellular growth control. While recent studies have shown that the activation state of RAS can be controlled by lysine ubiquitylation and acetylation, the existence of lysine methylation of the RAS superfamily GTPases remains unexplored. In contrast to acetylation, methylation does not alter the side chain charge and it has been challenging to deduce its impact on protein structure by conventional amino acid substitutions. Herein, we investigate lysine methylation on RAS and RAS-related GTPases. We developed GoMADScan (Go language-based Modification Associated Database Scanner), a new user-friendly application that scans and extracts posttranslationally modified peptides from databases. The GoMADScan search on PhosphoSitePlus databases identified methylation of conserved lysine residues in the core GTPase domain of RAS superfamily GTPases, including residues corresponding to RAS Lys-5, Lys-16, and Lys-117. To follow up on these observations, we immunoprecipitated endogenous RAS from HEK293T cells, conducted mass spectrometric analysis and found that RAS residues, Lys-5 and Lys-147, undergo dimethylation and monomethylation, respectively. Since mutations of Lys-5 have been found in cancers and RASopathies, we set up molecular dynamics (MD) simulations to assess the putative impact of Lys-5 dimethylation on RAS structure. Results from our MD analyses predict that dimethylation of Lys-5 does not significantly alter RAS conformation, suggesting that Lys-5 methylation may alter existing protein interactions or create a docking site to foster new interactions. Taken together, our findings uncover the existence of lysine methylation as a novel posttranslational modification associated with RAS and the RAS superfamily GTPases, and putative impact of Lys-5 dimethylation on RAS structure.

## Introduction

The small GTPase RAS is a signaling switch, cycling between active GTP- and inactive GDP-bound states. As dysregulation of RAS activity promotes cancer [1–6] and developmental disorders [7–10], wide-scale efforts are in place to generate agents that antagonize aberrant RAS function. Although development of direct inhibitors of RAS has historically proven challenging [11–15], compounds that target a specific oncogenic mutant (KRAS G12C) [16–18] are now in phase I clinic trials (Clinical Trial number: NCT03600883, NCT03785249). While these inhibitors appear quite promising, they target one out of many possible oncogenic RAS mutants. Moreover, oncogenic RAS mutants are populated in the active GTP-bound state, and these covalent inhibitors react with the less active GDP-bound state. Hence, understanding novel mechanisms of RAS regulation may prove helpful in identifying new therapeutic approaches for targeting RAS-driven tumors and developmental disorders.

RAS proteins contain a core guanine nucleotide binding (G)-domain that consists of five conserved G boxes [19,20]. These G boxes are the basis of the high affinity and specificity of RAS proteins for guanine nucleotides (GDP and GTP). Binding of either GDP or GTP promotes distinct conformational changes in two key regions, termed switch I and II. In an unstimulated cell, cellular RAS is populated in the ‘inactive’ GDP-bound conformation, despite the high GTP/GDP intracellular ratio. Guanine nucleotide Exchange Factors (GEFs) bind and upregulate RAS activity by promoting nucleotide exchange to facilitate association of the more abundant cytosolic GTP, whereas RAS GTPase activating proteins (GAPs) bind to RAS and accelerate the intrinsic rate of GTP hydrolysis to downregulate RAS activity [21–23]. The active RAS GTP-bound conformation of the two switch regions is recognized by effector targets and culminates in downstream signaling.

Oncogenic mutations in RAS promote dysregulated cell proliferation [1–10]. While mammalian cells encode four closely related RAS proteins, (HRAS, NRAS, and KRAS4A/4B), most oncogenic mutations occur in KRAS [10,11,24–26]. Hot spot mutations found at glycine-12 and glycine-13 in the G1 box, and less frequently at glutamine-61 in the G3 box, populate the active GTP-bound form of RAS by interfering with GAP-mediated GTP hydrolysis. A subset of RAS mutations in G4 and G5 boxes leads to RAS activation by promoting nucleotide exchange [27–30]. For example, the G5 box, or the S-A-X motif, contains residues that interact with the guanine moiety and is required for selective and high affinity binding of RAS to guanine nucleotides. Mutations of the alanine residue, Ala-146, in the S-A-X motif have been found in cancers and developmental disorders and promotes activation of RAS by increasing GDP exchange and GTP loading [28,30–35]. Residue X in the S-A-X motif corresponds to Lys-147 in RAS, and is ~ 55% conserved in RAS proteins across species and in a number of RAS-superfamily GTPases including RHOA, RAP, RAL, RAB, RHEB and RAN [19]. Oncogenic mutations are also found in residues outside the G-box. Lys-5 shows about 70% conservation within the RAS superfamily GTPases and lies within the amino terminus of RAS [19]. While the role of Lys-5 is not well understood, two missense mutations (K5N and K5E) have been identified in cancers [31–33] and in patients with Noonan, Cardiofaciocutaneous, and Costello syndromes [34–37], suggesting that these are activating mutations.

Precise control of RAS function is essential for cellular growth control. Unlike genetic lesions, reversible post-translational modifications (PTMs) provide a distinct mechanism to regulate RAS activity and function. In fact, RAS GTPases are regulated by a number of lysine PTMs, including acetylation and ubiquitylation [38,39]. While polyubiquitylation of RAS promotes proteasome dependent degradation [40–43], lysine monoubiquitylation of KRAS at lysine 147 in the G5 box, has been shown to upregulate KRAS activity by impairing GAP-mediated GTP hydrolysis [44,45]. Lysine 147 is also a site of KRAS acetylation [46,47], however, the role of lysine acetylation at this site is unclear. It is becoming increasingly clear that lysine acetylation can alter protein function outside its well-documented role in histone-mediated transcriptional regulation [48–50].

In contrast to lysine acetylation, the role of lysine methylation beyond chromatin regulation is less well-characterized, despite its earlier discovery in *Salmonella typhimurium* flagellin protein in 1959 [51]. Lysine modifications are more diverse than acetylation and can involve the transfer of one, two or three methyl groups to the ε-amine of a lysine side chain. Lysine methylation been identified in a host of abundant cellular proteins, including histones [52], cytochrome c [53], ribosomal proteins [54,55], myosin [56], and EF-Tu [57,58], suggesting a fundamental role in eukaryotes and prokaryotes. However, identification of this PTM has long relied on analyses that require large amounts of the target protein, such as Edman sequencing, radiolabeled assays or immunoblotting of the targeted site. Furthermore, it has been challenging to deduce the role of lysine methylation, since, as opposed to lysine acetylation, methylation does not alter the lysine side chain ε-amine charge and thus amino acid substitutions do not adequately mimic lysine methylation. Chemical biology methods have been developed to chemically introduce methylated lysine into proteins *in vitro* [59,60], but are limited by the requirement for recombinant protein, expertise in chemistry and yield of the desired modification. Consequently, our understanding of the biological significance of this PTM has been limited to a few proteins, including histones [61–64] and p53 [65–68].

In this study, we explored a possible new layer of small GTPase regulation by lysine methylation using a new application, GoMADScan, in combination with mass spectrometry approaches. Our MS analysis identified novel methylation sites at conserved lysines, Lys-5 and Lys-147, within the core RAS GTPase domain. Importantly, GoMADScan also identified Lys-5 methylation in the RAN GTPase, suggesting lysine methylation at this site may represent a conserved mechanisms of regulation. Results from molecular dynamics (MD) simulations indicate that Lys-5 dimethylation does not significantly alter RAS structure or dynamics. Rather, we posit that lysine methylation at this site alters existing interactions or creates a binding interface to foster new interactions. Together, this study identifies for the first time using MS analyses, a novel layer of RAS modification. We also highlight the use of GoMADScan and MD simulations as a systematic and versatile approach to extract lysine methylation sites from databases and assess their potential impact on the activity of small GTPases.

## Material and methods

### Materials

Anti-RAS (#05–516) was obtained from EMD/Calbiochem; rat monoclonal anti-RAS (Y13-238)-conjugated agarose (#sc-34 AC) from Santa Cruz; anti-Flag (M2) antibody and bovine serum albumin (BSA) (Fraction V) were purchased from Sigma-Aldrich. Bis-Tris gels were obtained from Invitrogen.

### Mass spectrometry analysis

Endogenous RAS proteins were immunoprecipitated with monoclonal rat anti-RAS antibody (238) from HEK293T and subjected to SDS-PAGE for mass spectrometry analysis as in [44]. MS/MS spectra were acquired using collision-induced dissociation. They were searched against the reversed and concatenated Swiss-Prot protein database (v. 55.8, UniProt) with a fixed modification for methionine methylation (+15.99490) and the variable modifications for monomethylation (+14.0156), dimethylation (+28.0106) and trimethylation (+42.0106) using the Sequest algorithm associated with the Proteomics Browser Software (Thermo Scientific, San Jose, CA). RAS peptides were identified by database scoring. Peptides modified by methylation were validated manually to be sure that all b- and y- series ions were consistent with the modified residue. Additional validation was performed using GraphMod software in Proteomics Browser Software (Thermo Scientific, San Jose, CA). The peptide false discovery rate was less than 1.5% based on reversed database hits.

### GoMADScan version 1.0

GoMADScan (version 1.0) is written in the Go programming language, and can run on multi-platforms where the GTK library is available. We tested GoMADScan running in OS X 10.11 with Go 1.3 and 10.14 with Go 1.8. Briefly, GoMADScan is designed to do simple keyword searches on delimiter-separated values (DSV) files with some drop-down lists and a scale bar to graphically change search conditions. This type of file format is widely used in biological databases. In particular, PhosphoSitePlus archives the large DSV-based dataset to extract protein modification sites. Thus, we applied GoMADScan for the PhosphoSitePlus dataset and used keywords to search for modifications within RAS superfamily GTPases [19]. GoMADScan is freely available at https://github.com/carushi/GoMADScan.

### Database search for modified RAS superfamily GTPases

Methylated peptides were downloaded from PhosphoSitePlus(R) (http://www.phosphosite.org) [69,70]. Lysine methylation sites were extracted using GoMADScan using name ‘matches’ within the gene list (Table 1). For scanning, we used the gene names adapted from [19] as below. After this curation, 72 methylation sites were identified in 40 RAS genes out of a total 19,745 methylation sites as of March 3th, 2019. For scanning, we used the gene names adapted from [19] and included their synonyms as the following:

**Table 1:** The list of gene symbol of Ras family.

AGS1	ARL10B	GBTS1	NY-MEL-1	RAB29	RAB5B	RAN	RHEBL1	RIF	WRCH-2
APMCF1	ARL10C	GEM	R-RAS	RAB2A	RAB5C	RAP1A	RHES	RIG	WTH3
ARD1	ARL11	GES	R-RAS2	RAB2B	RAB5CL	RAP1B	RHO6	RIN	YL8
ARF1	ARL2	GIE1	R-RAS3	RAB30	RAB6A	RAP2A	RHO7	RIS	
ARF3	ARL2L1	GIE2	RAB10	RAB31	RAB6B	RAP2B	RHO8	RIT1	
ARF4	ARL3	GOV	RAB11A	RAB32	RAB6C	RAP2C	RHOA	RIT2	
ARF4L	ARL4	H-RAS	RAB11B	RAB33A	RAB7A	RAR	RHOB	RND1	
ARF5	ARL5	H-RASIDX	RAB12	RAB33B	RAB7B	RAR-2	RHOBTB1	RND2	
ARF6	ARL6	H-RAY	RAB13	RAB34	RAB7L1	RAR2A	RHOBTB2	RND3	
ARFD1	ARL7	H-YPT3	RAB14	RAB35	RAB8A	RAR3	RHOC	RNF46	
ARFRP1	ARL8	HRAS	RAB15	RAB36	RAB8B	RARL	RHOD	ROC1	
ARFRP2	ARL9	HRAS2	RAB16	RAB37	RAB9A	RASD1	RHOE	ROC2	
ARHA	ARLTS1	HRASP	RAB17	RAB38	RAB9B	RASD2	RHOF	RRAD	
ARHB	ARP	HSPC137	RAB18	RAB39	RAB9L	RASEF	RHOG	RRP22	
ARHC	BBS3	KIR	RAB19	RAB39A	RABL	RASL10A	RHOH	SAR1A	
ARHD	CDC42	KRAS2A	RAB19B	RAB39B	RABL2A	RASL10B	RHOH12	SAR1B	
ARHE	CDC42HS	KRAS2B	RAB1A	RAB3A	RABL2B	RASL11A	RHOH6	SARA1	
ARHF	CDC42L1	KREV-1	RAB1B	RAB3B	RABL3	RASL11B	RHOH9	SARA2	
ARHG	CHP	LAK	RAB1C	RAB3C	RABL4	RASL12	RHOHP1	SEC4L	
ARHH	CMRD	LOC339231	RAB20	RAB3D	RABL5.	RASL7A	RHOI	SMGP21	
ARHH	D2-2	LOC401884	RAB21	RAB40A	RABS10	RASL7B	RHOJ	SRPRB	
ARHI	DBC2	M-RAS	RAB22A	RAB40B	RAC1	RASL8C	RHON	TC10	
ARHJ	DEXRAS	MASRA2	RAB22B	RAB40C	RAC2	RAY	RHOQ	TC10BETA	
ARHN	DI-RAS1	MEL	RAB23	RAB41	RAC3	RAYL	RHOT	TC21	
ARHQ	DI-RAS2	MIRO-1	RAB24	RAB42	RAD	REM1	RHOT1	TC25	
ARHS	E-RAS	MIRO-2	RAB25	RAB43	RAD3D	REM2	RHOT2	TCL	
ARHU	FBP	N-RAS	RAB26	RAB45	RAH	REM3	RHOU	TEM2	
ARHV	FLJ22595	NKIRAS1	RAB27A	RAB4A	RALA	RERG	RHOV	TRIM23	
ARL1	FLJ22655	NKIRAS2	RAB27B	RAB4B	RALB	RHEB1	RIBA	TTF	
ARL10A	G25K	NOEY2	RAB28	RAB5A	RAM	RHEB2	RIBB	WRCH-1	

### Molecular dynamic simulations

The starting structures for the GDP- and GTP-bound forms of KRAS were PDB ID 4lpk [16] and PDB ID 6god [71], respectively. The C-terminal helix of the GDP-bound structure was extended by 6 residues using PyMOL [The PyMOL Molecular Graphics System, Version 2.3 Schrödinger, LLC] for consistency with the GTP-bound structure. The GNP residue in 6god was converted to GTP by converting the N3B atom into O3B. The N-terminal serine from 6god was mutated to methionine for consistency with 4lpk. Lysine 5 in KRAS was dimethylated using MLY from 3mp6 [72] as a template. Molecular dynamics (MD) simulations were completed in triplicate for each of the four configurations (GDP- and GTP-bound with and without dimethylated Lys-5) for a total of twelve simulations. MD simulations were conducted using the CUDA version of PMEMD [73] [74,75] from the Amber16 suite of programs (UCSF) [76]. Protein parameters were from the ff14SB force field [77], while the force field parameters for dimethylated lysine were adapted from [78]. Force field parameters for GDP and GTP were obtained from the Bryce Group Computation Biophysics and Drug Design Amber Parameter Database (www.pharmacy.manchester.ac.uk/bryce/amber) [79]. To initiate the simulations, RAS proteins were placed in a TIP3P octagonal water box extending 16.0 Å from the protein edge with Na+ counter ions included. Topology and starting files were generated using the tleap program. Electrostatic interactions were calculated using the particle-mesh Ewald method with a cutoff of 8 Å for non-bonded interactions. The SHAKE algorithm was applied to constrain bonds involving hydrogens. All MD simulation production runs were conducted under constant volume and constant temperature periodic boundary conditions with an Andersen thermostat. For equilibration, 10000 minimization steps were applied at the beginning, including 5000 steepest descent and 5000 conjugate gradient minimization steps. The system was then heated from 0K to 300K using 500 ps of constant volume dynamics. This was followed by 500 ps constant pressure to reach a density of 1g/cm^3^ for the entire system. Production simulation replicates were each run for a total length of 200 ns with a 2 fs timestep, recording snapshots every 10 ps. Based on the analysis of the backbone Cα root-mean-square-deviation (RMSD), the first 10 ns of the trajectories were excluded from the further analysis as the systems equilibration time. The trajectories were then subjected to distance-based clustering analysis using GROMACS 2018 [80] clustering algorithm [81]. The distance cutoff for each system was selected by analyzing the corresponding RMSD distributions with respect to the starting structures. The cutoff was then selected as the value corresponding to the peak of the distribution. The centroids of most populated clusters were then selected as the representative structures of the complexes. Trajectories were analyzed using a density-based clustering algorithm. The inputs of the analysis were 25 points as the minimum number required to form a cluster and distance cutoff of 0.7 between points for forming a cluster. The appropriate distance cutoff was analyzed using a K-dist plot which shows the Kth farthest distance for each point, sorted by decreasing distance. The minimum number of points required to form a cluster is the value of K used in the K-dist plot.

### GST-cRAF1 RAS binding domain (RBD) pull-down assay

Flag-His- (FH)-tagged KRAS4B mutants were generated by the standard polymerase chain reaction (PCR) method and subcloned into pCMV2. RAS activation was measured as described previously[44,82]. Briefly, FH-KRAS4B were transfected with Fugene 6 to HEK293T cells. Twenty four hours after the transfection, cells were rinsed with cold PBS and lysed with Buffer A; 0.5%NP-40, 40 mM HEPES [pH 7.4], 150 mM NaCl, 10% glycerol, 1 mM DTT, 1 μg/ml leupeptin, 2 μg/ml aprotinin, 1 μg/ml pepstatin A, 100 μM AEBSF, and Halt phosphatase inhibitor cocktail (Thermo Scientific). The soluble fraction of cell lysates were isolated by centrifugation at 13,000 rpm for 10 min and incubated with 10 μg of GSH-Sepharose bound GST-RBD in the presence of 1 mg/ml BSA for 30 min. The pull-downed proteins were washed three times with Buffer A and subjected to SDS-PAGE and western blot analysis.

## Results

### Methylation site scanning identifies lysine methylation in the G domain of RAS superfamily GTPases

**Fig 1:**
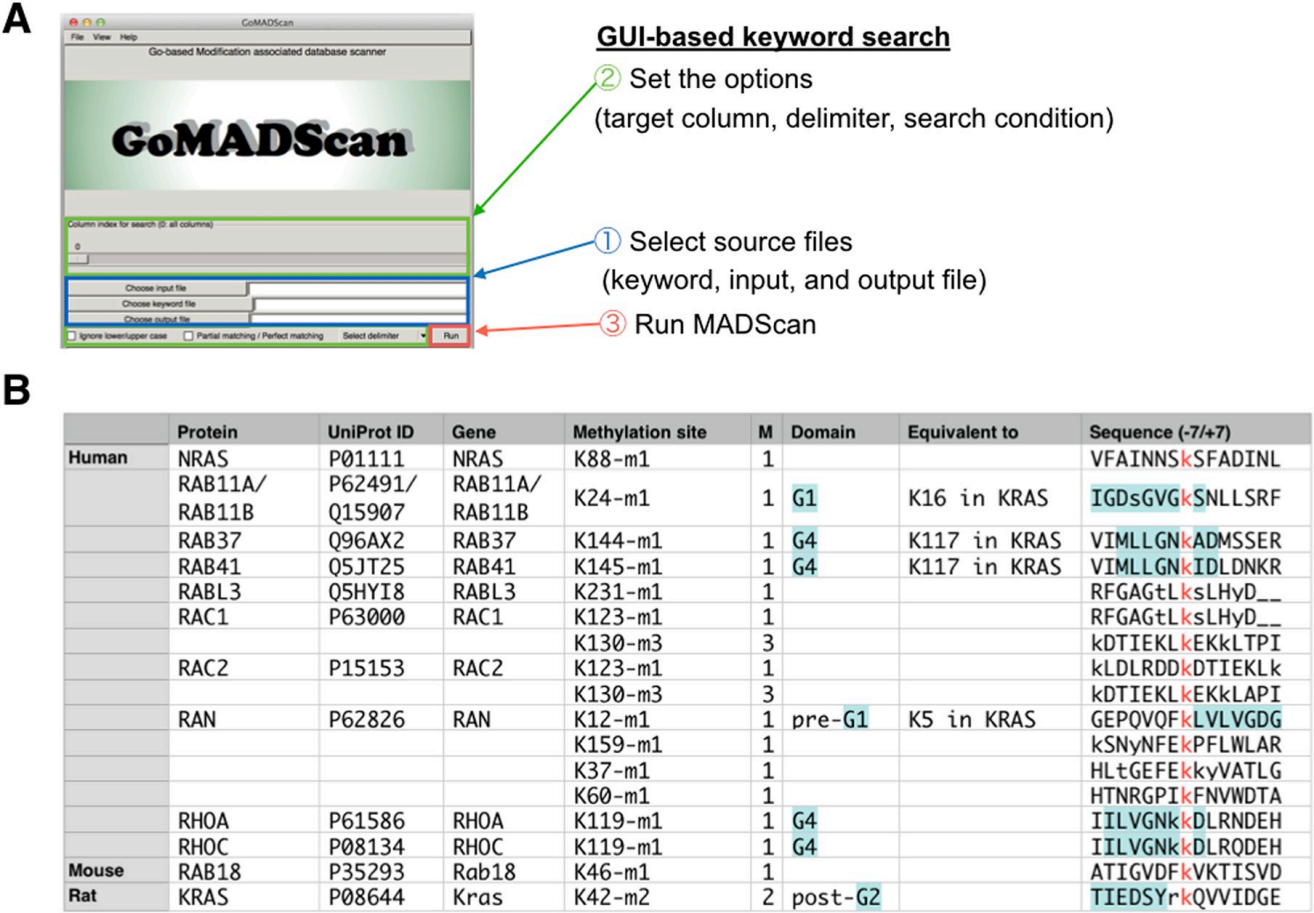
Identification of lysine methylation in the core GTPase domain of several RAS superfamily GTPases by the GoMADScan. **(A)** GoMADScan is a free GUI-based search application available at https://github.com/carushi/GoMADScan that scans to extract posttranslationally modified peptides from databases. The screenshot of GoMADScan is shown in left. GoMADScan analysis consists of three simple steps: Step 1- input file such as methylation- or ubiquitylation-site dataset. For the present work, the PhosphositePlus(R) database was used. Step 2—select a keyword file for the proteins of interest. Keywords can include any features such as gene name, motif sequence, or modification type. At Step 3, GoMADScan extracts a part of database containing the specified keywords. Instruction video is available at https://www.youtube.com/watch?v=PCXOWjk9d_E **(B)** GoMADScan identification of methylated lysine peptides associated with RAS superfamily members.

To explore whether lysine methylation occurs in RAS related proteins by database search, we developed GoMADScan (Go language-based Modification Associated Database Scanner: https://github.com/carushi/GoMADScan). GoMADScan is a prototype scanning software for complex data mining to extract post-translationally modified peptides based on type of modification, peptide motif and protein domain (Fig 1). We applied GoMADScan to extract methylated peptides associated with RAS superfamily GTPases from the protein modification database PhosphoSitePlus(R) (http://www.phosphosite.org) [69,70], and identified 18 lysine methylated peptides in 13 small GTPases (Fig 1B). The RAN GTPase (Ras-related small nuclear protein; regulator of the nucleocytoplasmic transport) as well as RAB GTPases (involved in membrane trafficking), were among the small GTPases identified. While it is possible that monomethylation is specific to RAN and RAB GTPases, RAN GTPases may have been identified because of their high cellular abundance (~ 100 μM, 0.36% of total cellular proteins [74, 75]. Similarly, RAB is the largest family among the RAS superfamily GTPases, consisting of ~60 members in mammalian [83–85]. In contrast, pan-RAS concentrations in a HeLa cells have been reported as 0.4 μM [86], which is ~250-fold less abundant than RAN.

### Tandem mass spectrometry analysis of endogenous RAS identifies Lys-5 and Lys-147 as methylation sites

**Fig 2:**
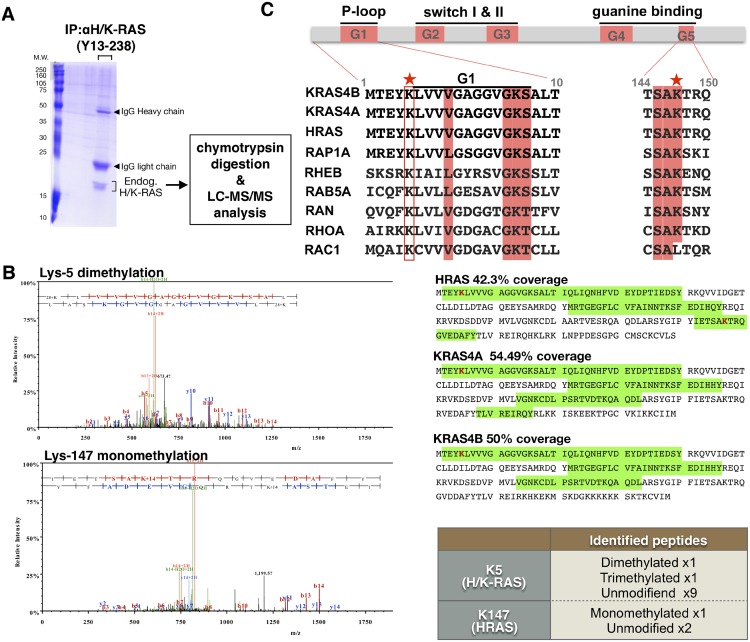
Lysine methylation sites identified in endogenous RAS. **(A)** Detection of Lys-5 and Lys-147 methylation in endogenous RAS proteins. HEK293T cells lysate was immunoprecipitated with anti-RAS antibody (Y13-238) and subjected to SDS-PAGE and Coomasie Brilliant Blue (CBB) staining. The corresponding band for RAS was purified from the gel and digested with chymotrypsin for LC-MS/MS analysis. **(B)** For microcapillary/tandem mass spectrometry (LC-MS/MS) experiments, purified ubiquitylated RAS bands were excised from analogous Coomassie blue-stained gels, digested with trypsin, and analyzed by LC-MS/MS. The MS/MS spectrum for the RAS peptides containing methylated lysine at the indicated position acquired through CID using a hybrid linear ion trap-Orbitrap mass spectrometer. The right sections show the detected regions by the mass spectrometry (green highlight) and the coverage (%). The identified methylated lysine and the number of detected peptides are shonw in lower right. **(C)** Sequence alignment highlighting the conserved motif in G-box for a subset of RAS superfamily GTPases (lower). The lysine methylation sites are marked with a red star. Color shaded boxes indicate conserved amino acids within in the G-box. The red line box shows sequence conservation of RAS residues. Lys-5 is adjacent to the G1 box.

To examine whether RAS undergoes lysine modification, endogenous RAS proteins were immunoprecipitated with a monoclonal anti-RAS antibody, Y13-238, from HEK293T cells. This antibody reacts with HRAS and KRAS, but not with NRAS [87,88]. After SDS-PAGE and Coomassie blue staining, the corresponding RAS protein band was isolated (Fig 2A). The immunoprecipitated, endogenous RAS protein was digested with chymotrypsin followed by liquid chromatography-tandem mass spectrometric analysis (LC-MS/MS). The LC-MS/MS analysis identified dimethylation at Lys-5 and monomethylation at Lys-147 in RAS (Fig 2B). As the methylated Lys-5 peptide sequence is identical in all RAS isotypes (Fig 2C), we were unable to deduce which RAS isotype (*i*.*e*. HRAS or KRAS), or isoform (*i*.*e*. KRAS 4A or 4B), undergoes methylation at Lys-5. However, we were able to determine isotype and isoform specificity for Lys-147, as the Lys-147 peptide sequence is unique to the HRAS (Fig 2C). Given that our previous work has shown that amino acid substitutions at Lys-147 to alanine, cysteine or leucine did not alter RAS activity [44,45], we predict that methylation of Lys-147 may not alter RAS structure. GoMADScan did not detect methylation at the equivalent position to Lys-147 in other small GTPases.

In contrast, Lys-5 methylation occurs in the RAN GTPase, suggesting that methylation at this site may represent a conserved mechanism of regulation. As Lys-5 mutations are found in cancer and RASopathies, we employed molecular dynamics (MD) simulations to investigate the putative impact of Lys-5 dimethylation on RAS structure and dynamics.

### Molecular dynamic simulations predict that dimethylation of Lys-5 in RAS does not significantly alter RAS structure and dynamics

Regulation of non-histone proteins by lysine methylation is currently an underdeveloped field, and there is no systemic approach to deduce the consequences of the lysine methylation at specific site(s). Methylation does not change the overall charge of the lysine or arginine side chain, and amino acid substitutions are unable to function as mimetics. Thus, to investigate the possible role of Lys-5 methylation in RAS function, we pursued structure-based analyses.

**Fig 3:**
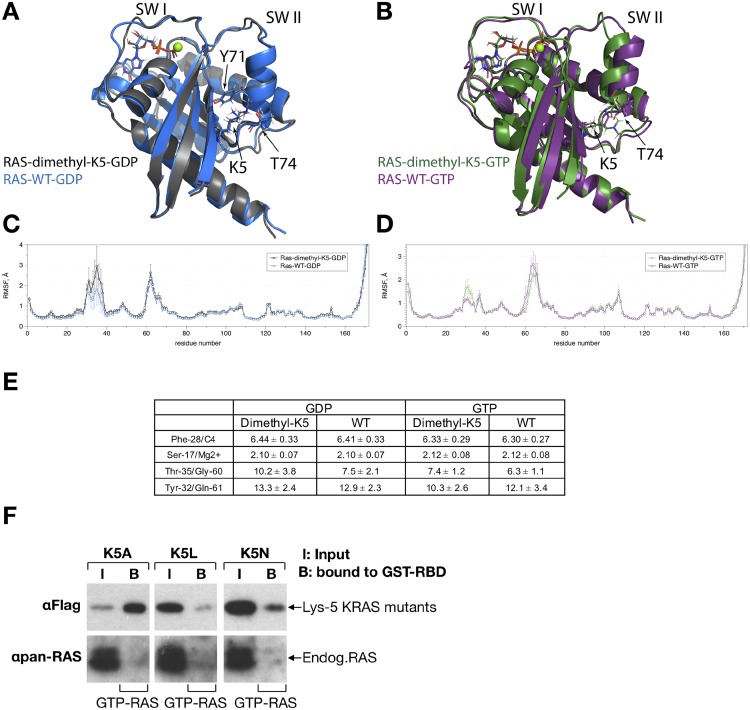
Ribbon depiction comparing dimethylated Lys-5 KRAS to that of WT KRAS obtained from three 200 ns MD simulations. **(A)** Structural overlay of GDP-bound WT KRAS (blue, population ~83%) with GDP-bound dimethylated Lys-5 KRAS (black, population ~76%) and **(B)** GTP-bound WT (purple, population 86%) and GTP-bound dimethylated Lys-5 KRAS (green, population ~84%). Dimethylation of Lys-5 does not significantly affect packing interactions of Lys-5 with Y71 and T74 sidechains in the GDP-bound state or with T74 in the GTP-bound state. Average residue RMSF values and their standard errors obtained from three 200 ns MD trajectories comparing dimethylated Lys-5 (black) and WT (blue) KRAS for both **(C)** GDP- and **(D)** GTP-bound states. **(E)** Distances for key RAS/nucleotide interactions for unmodified and dimethylated RAS in both GDP- and GTP-bound states. Comparison shows similar distances, indicating proper nucleotide and magnesium association. These distances include the Cγ of Phe-28 to C4 of the guanine indole ring and Oγ of Ser-17 to Mg^2+^. All distances are calculated from three 200 ns MD trajectories for each system. Two distinct, key average distances between the two switch (Switch I and Switch II) regions are also compared, for wild type and Lys-5 dimethylated KRAS. Dimethylated Lys-5 KRAS does not significantly alter switch distances in either the GDP or GTP-bound states. Distances between O of Thr-35 and N of Gly-60 and between Cζ of Tyr-32 and Cδ of Gln-61 are shown. **(F)** Mutation of Lys-5 to Ala, Leu or Asn affects RAS activity. Flag-His tagged KRAS mutants were expressed in HEK293T cells, and their activation level analyzed by GST-RBD pull-down of RAS (see Materials and methods). Western blots with anti-Flag and anti-pan RAS antibodies reveal the relative activation levels of the KRAS mutants relative to endogenous RAS, respectively.

Lys-5 is a highly conserved residue that lies within beta strand 1 (β1) of RAS and is adjacent to the G1 box (Fig 3A). While this residue does not appear to form direct interactions with the bound nucleotide in either the GDP or GTP-bound states, the aliphatic sidechain of Lys-5 does interacts with the aromatic sidechain of Tyr-71 and the sidechain of Thr-74 in switch II (PDB: 1CRQ) in the GDP bound state. In the GTP-bound state, the Lys-5 aliphatic sidechain packs against the sidechain of Thr-74 at the end of switch II (Fig 3B). In the crystal of the RAS-SOS complex, Tyr-71 of RAS forms a hydrogen bond with Tyr-712 of the RASGEF, SOS (PDB: 1NVW). Tyrosine 74 is also located at the RAS interface with PI3 Kinase-γ (PDB: 1HE8). Hence, modulation of these interactions by lysine methylation might potentially alter GEF and effector interactions.

In an effort to predict the effects of dimethylation on RAS structure and dynamics, we employed MD simulations. Specifically, we evaluated whether dimethylation of RAS at Lys-5 alters the overall conformation, key interactions between RAS and its bound nucleotide, as well as structural and dynamic properties of the RAS switch regions. We conducted 200 ns MD simulations in triplicate for each of the four configurations (GDP- and GTP-bound with and without dimethylated Lys-5). To assess whether RAS-nucleotide interactions (GDP or GTP) are altered by Lys-5 dimethylation, key distances between KRAS residues and the bound nucleotide (GDP and GTP) (Fig 3E) were examined, as partial disruption of guanine nucleotide binding interactions could enhance nucleotide cycling and lead to GTP loading and activation in cells. We evaluated two key distances critical for guanine nucleotide binding, including the distance between Phe-28 Cγ and the guanine indole C4 atom as well as the distance between Ser-17 and Mg^2+^. As shown in Fig 3E, these RAS-nucleotide distances are similar for both non-modified and dimethylated Lys-5 KRAS in both the GDP and GTP-bound states. This analysis suggests that Lys-5 dimethylation does not significantly alter nucleotide binding.

We next assessed whether Lys-5 dimethylation alters the overall structure of KRAS. We first generated a structural superposition from three 200 ns MD trajectories (Fig 3) for both the GDP- and GTP-bound states of dimethylated Lys-5 KRAS and compared to unmodified KRAS. As observed in Fig 3A and 3B, the structures of wild type KRAS in both the GDP and GTP bound states overlay well with the corresponding structures of Lys-5 dimethylated KRAS. The average distances between the two switch regions were also evaluated. The conformation of the switch regions is dependent on the nucleotide bound state, with the GTP-bound conformational ensemble representing the active state. Adopting this active state confers higher affinity binding to downstream effectors. Key distances between the switch regions, specifically, O of Thr-35 in Switch I and N of Gly-60 in Switch II and between Cζ of Tyr-32 (Switch I) and CD of Gln-61 (Switch II) were measured. Overall, these distances between WT and dimethylated Lys-5 were found to be within experimental error for both GDP- and GTP-bound structures (Fig 3E). Results from these analyses indicate that Lys-5 dimethylation does not significantly affect the average structure of the RAS switch regions in both the GTP and GDP-bound states. As the switch regions are dynamic, we also calculated the average residue root mean square fluctuation (RMSF) values and their standard errors from the MD trajectories of the triplicates. As expected, we observe significant fluctuations in the switch regions, however (Fig 3C and 3D), the RMSF differences indicate that Lys-5 dimethylation does not significantly alter RAS dynamics in both GDP- and GTP-bound states of RAS. Taken together, our simulations predict that KRAS Lys-5 dimethylation maintains intrinsic nucleotide binding, conformation and dynamics. Hence, rather than altering RAS structure and nucleotide cycling, Lys-5 dimethylation may alter/promote new protein interactions or possibly alter interactions with the membrane [89].

While our MD simulations predict that the intrinsic nucleotide cycling properties of isolated KRAS are retained, we wanted to assess whether perturbation at Lys-5 alters RAS activity in cells. We expressed three KRAS mutants, K5A, K5L and K5N in HEK293T cells and employed a pull-down assay to probe for activated GTP-bound RAS, using the RAS binding domain (RBD) of human cRAF1 kinase fused to GST [90–92]. Consistent with previous findings, endogenous RAS is populated in the GDP state [44,90–92]. Strikingly, all 3 mutants at Lys-5 showed elevated binding to the GST-RAF RBD, suggesting that mutations at this position enhance cellular RAS activity (Fig 3F). These findings are consistent with previous observations that the activating RAS K5N mutant retains similar nucleotide cycling and RAF-RBD affinity *in vitro*, yet intriguingly shows enhanced RAS-RAF association and MAPK signaling [29,89]. Notably, both activating RAS K5N and K5E mutations are found in cancers [31–33] and in RASopathies [34–37]. However, it is unclear how these three mutations at Lys-5 promote RAS activation in cells. Similar to previous findings that the activating RAS K5N mutant does not disrupt nucleotide cycling, we predict that dimethylation at this site retains nucleotide binding properties. As these activating Lys-5 mutations may not fully recapitulate the effects of dimethylation, it remains unclear whether Lys-5 dimethylation will promote RAS activation at the cellular level.

## Discussion

In the present work, we have identified methylation at Lys-5 and Lys-147 as a novel post-translational modification in endogenous RAS. As our GoMADScan identified methylation at the equivalent site in the RAS-related GTPase, RAN, methylation of Lys-5 may have a conserved role in the regulation of these small GTPases.

**Fig 4:**
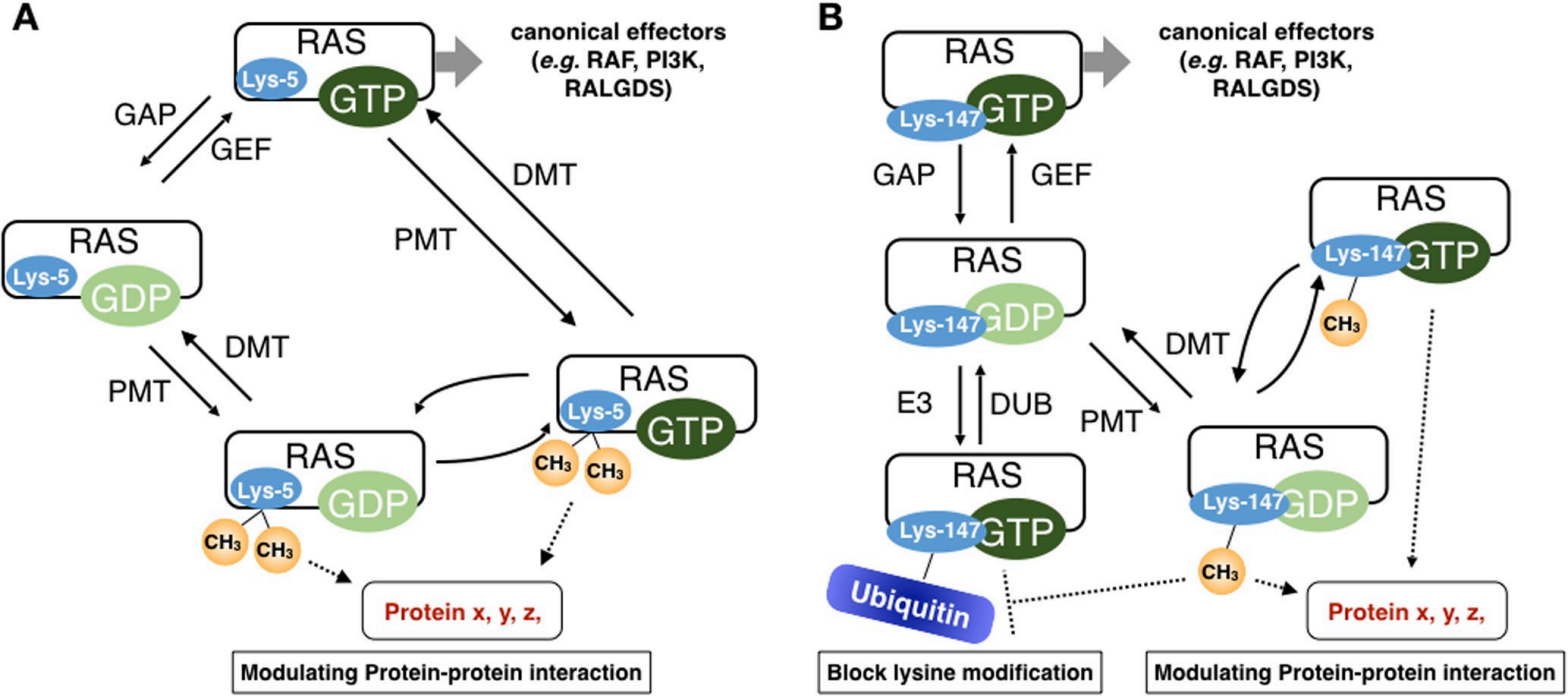
Putative roles of Lys-5 and Lys-147 methylation. **(A)** Model of RAS regulation by methylation at Lys-5. The population of active GTP- and inactive GDP-loaded RAS is regulated by GEFs and GAPs. RAS Lys-5 dimethylation occurs from unidentified protein methyl transferase(s) (PMT) and removed by demethylases (DMT). Lys-5 dimethylation does not alter RAS structure or dynamics. Rather, lysine dimethylation may alter known protein-protein or protein-membrane interactions or creates a new docking site for new interactants such as methyl-lysine binding proteins. **(B)** Model for RAS regulation by methylation at Lys-147. Methylation at Lys-147 may interfere with ubiquitylation and acetylation at this site. In parallel, Lys-147 methylation may alter existing interactions or create a new docking site to facilitate new interactions such methyl-lysine binding proteins, inducing non-canonical RAS signaling.

Lysine methylation on non-histone proteins remains a largely unexplored area. The studies from the lysine methylation on histones established that a lysine methylated peptide acts as a docking site for new protein-protein interactions [61–64]. However, it is less clear whether lysine methylation induces conformational changes in target proteins due to tools available for lysine methylation. Approach using amino acid substitutions is powerful but fail to mimic lysine methylation. Chemical introduction of methylated lysine into proteins *in vitro* [59,60] requires expertise in chemistry and the method is still not the widely available. These are the bottleneck factors that have limited our understanding of the biological significance of lysine methylation. In the present study, we have employed MD analysis, a versatile and established approach, to predict changes in RAS conformation in response to lysine methylation. Our MD analyses predict that the dimethylation of Lys-5 maintains nucleotide binding, structure and dynamics (proposed model is depicted in Fig 4A), suggesting that rather than altering intrinsic RAS structure and nucleotide cycling, dimethylation may alter interactions or create a new docking site for an as of yet unidentified interaction(s). Though additional studies are needed, the MD simulation implicate a new layer of regulation of RAS structure by dimethylation of Lys-5.

Methylation of Lys-147, a conserved lysine in G5 box, was also detected by our LC-MS/MS analysis. A subset of RAS mutations in G5 box also activates RAS by promoting nucleotide exchange [28,30,93]. However, oncogenic mutations have not been identified Lys-147 and amino acid substitutions at Lys-147 to alanine, cysteine or leucine do not significantly alter RAS activity [44,45]. Thus, it is possible that methylation at Lys-147 retains the key interactions of the S-A-X motif with guanine nucleotides, but creates a new docking site for methyl-lysine binding proteins, or modulates RAS ubiquitylation [44,45,94], acetylation [46,47], or other potential lysine modifications at this site (proposed model is depicted in Fig 4B).

It is important to note that methylation may occur at other sites in RAS as our MS-based analysis does not fully cover the entire RAS protein sequence (Fig 2B). This is due to the limitation of chymotrypsin for LC-MS/MS based detection as well as detection sensitivity for several peptide fragments. While GoMADScan allows rapid identification of other conserved lysine methylation sites in RAS superfamily GTPases within the large databases (Fig 1A), our MS did not identify methylation at Lys-117. It is possible that use of other digestion enzymes, such as trypsin, may increase the detection of the methylation at these sites, or that the sites of lysine modification are unique for the GTPase. Clarification of the RAS isotype(s) that undergo Lys-5 methylation will provide helpful information in future work.

Together, our data, for the first time to report that endogenous RAS undergoes lysine methylation at two conserved lysine residues within the core GTPase domain. As our MD analyses predict that methylation at Lys-5 maintains intrinsic RAS structure and nucleotide cycling, we postulate that methylation may alter protein-protein interactions and RAS signaling. Given that the lysine methylome is rapidly expanding, combining GoMADScan and MD simulation should facilitate studies of lysine methylation of RAS superfamily GTPases and possibly other enzymes.
