# Correction: Identification of lysine methylation in the core GTPase domain by GoMADScan

**DOI:** 10.1371/journal.pone.0224443

**Published:** 2019-10-22

**Authors:** Hirofumi Yoshino, Guowei Yin, Risa Kawaguchi, Konstantin I. Popov, Brenda Temple, Mika Sasaki, Satoshi Kofuji, Kara Wolfe, Kaori Kofuji, Koichi Okumura, Jaskirat Randhawa, Akshiv Malhotra, Nazanin Majd, Yoshiki Ikeda, Hiroko Shimada, Emily Rose Kahoud, Sasson Haviv, Shigeki Iwase, John M. Asara, Sharon L. Campbell, Atsuo T. Sasaki

The title of this article is incomplete. The correct title is: Identification of lysine methylation in the core RAS GTPase domain by GoMADScan. The correct citation is Yoshino H, Yin G, Kawaguchi R, Popov KI, Temple B, Sasaki M, et al. (2019) Identification of lysine methylation in the core RAS GTPase domain by GoMADScan. PLoS ONE 14(8): e0219436. https://doi.org/10.1371/journal.pone.0219436
